# Three-dimensional reconstruction of systematic histological sections: application to observations on palatal shelf elevation

**DOI:** 10.1038/s41368-021-00122-8

**Published:** 2021-05-26

**Authors:** Weilong Liu, Xiaoming Wang, Yinuo Wang, Yahong Wang, Jing Zhang, Bing Shi, Chenghao Li

**Affiliations:** 1 State Key Laboratory of Oral Diseases & National Clinical Research Center for Oral Diseases & Department of Cleft Lip and Palate Surgery, West China School of Stomatology, Sichuan University, Chengdu, China; 2 College of Electrical Engineering and information Technology, Sichuan University, Chengdu, China

**Keywords:** Developmental biology, Genetics

## Abstract

Normal mammalian secondary palate development undergoes a series of processes, including palatal shelf (PS) growth, elevation, adhesion and fusion, and palatal bone formation. It has been estimated that more than 90% of isolated cleft palate is caused by defects associated with the elevation process. However, because of the rapidly completed elevation process, the entire process of elevation will never be easy to clarify. In this article, we present a novel method for three-dimensional (3D) reconstruction of thick tissue blocks from two-dimensional (2D) histological sections. We established multiplanar sections of the palate and tongue in coronal and sagittal directions, and further performed 3D reconstruction to observe the morphological interaction and connection between the two components prior to and during elevation. The method completes an imaging system for simultaneous morphological analysis of thick tissue samples using both synthetic and real data. The new method will provide a comprehensive picture of reorientation morphology and gene expression pattern during the palatal elevation process.

## Introduction

Cleft palate is a common craniofacial malformation in humans, affecting approximately one in every 700 live births worldwide. During mammalian palatogenesis, the secondary palatal shelf (PS) undergoes dramatic morphological changes to elevate from the vertical to horizontal direction and finally separate the oral and nasal cavities. Because of the rapid process, the exact molecular mechanism still remains elusive.^1–5^ Several hypotheses about the morphological changes have been considered for decades. Walker and Fraser proposed that the PS rapidly remodeled itself by bulging a new protrusion on the lingual wall and regression at the original tip, and described that the PS reorientation proceeded in a wave-liking manner from posterior to anterior.^1,4,6^ Coleman found that the anterior part of the PS was elevated by a swinging “flip-up” process along the AP (anterior–posterior) axis, while the posterior and middle parts were reoriented through an oozing “flow” remodeling mechanism.^1,7^ Although both these authors believed dramatic morphological remodeling occurred during elevation in the posterior palate, little was mentioned about the decisive factor for the distinct elevation pattern along the AP axis.^1,4,8^ Several investigators hypothesized that the intrinsic bulging or swelling force, generated by the absorption of water by hyaluronan and glycosaminoglycan, expanded the extracellular matrix (ECM) to remodel the PS and accomplish elevation.^1,3,8–10^ For the above reasons, and the fact that aglossia could also be accompanied by the normal palatogenesis,^11,12^ some researchers considered that the tongue was not important in assisting palatal elevation.^5^ However, some studies suggested that the lack of fetal tongue or mouth movement could also result in a delay in palatal elevation,^13,14^ implying the positive role of tongue in palatal elevation. Irrespective of the hypothesis, the tongue plays an important role in palatal elevation due to its key location in the oral and nasal cavities. However, because of the rapid process, it is difficult to capture the dramatic morphological changes of PSs as they conflicted with the tongue.

Two-dimensional (2D) histology studies using serial tissue sections have been extremely insightful in identifying and quantifying factors, such as the regional observation of a tissue sample’s morphology and intra- and extra-cellular structure, and cell proliferation and apoptosis, which may contribute to the development. However, 2D histology has limitations in providing a holistic picture of the problems occurring during mammalian palatogenesis and struggles to analyze the spatial and temporal changes in the general overview of morphology. Compared with a 2D section, a three-dimensional (3D) model can reflect the spatial information of the components of organs, which is widely used for ascertaining 3D architectural information.^15–18^ In palatal development, although previous studies reported the 3D structures of the palate and tongue at later stages using (micro computed tomography) micro-CT,^19^ the 3D architectural interaction between the palate and tongue during the earlier elevation procedure remains unknown. Here, we present a novel method for the 3D reconstruction of thick tissue blocks from 2D histological sections. We established multiplanar sections of the palate and tongue in the coronal and sagittal directions, and we further performed 3D) reconstruction to observe the morphological interaction and connection between the two components prior to and during elevation.

## Results

### The important anatomical structure of the palate and tongue before elevation

**Fig. 1:**
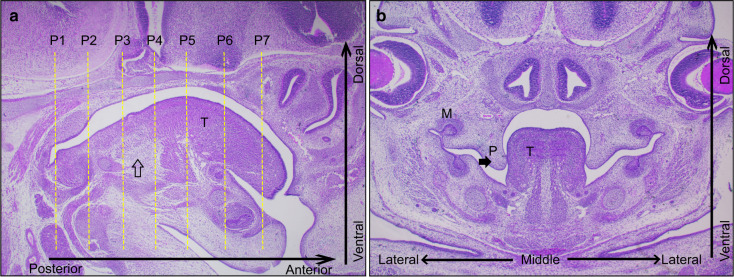
The early E13.5 embryo as an example. **a** Planes 1–7 were established at about 100 μm intervals along the anterior–posterior (AP) axis in the median sagittal view. P1 was set at the position where the PSs initially emerged, and P6 was the border where the tongue separated from the lingual frenulum. **b** The coronal view of the PS and tongue before elevation at early E13.5. The empty arrow indicated the genioglossus. P, palate; T, tongue; M, molar; M, medial; L, lateral

Current viewpoints emphasized that the middle and posterior parts of the PS undergo elevation by remodeling, while the anterior part just undergoes the flip-up process.^1,8^ Less attention has been paid to the morphological correlation and interaction between the tongue and PS. Here, to clarify the regional morphological characters of the PS and tongue in detail, we established 7 coronal planes along the AP axis (Planes 1–7 from posterior to anterior) by setting and sectioning embryos with the hindbrain parallel to the bottom of the paraffin-embedded box. We defined the section where the palatal shelves initially emerged as Plane 1 and selected the next plane at an interval of 15 sections, about 105 μm for the early and late E13.5 embryo (the E12.5 embryo was set at an interval of 10 sections, 70 μm) (Fig. 1). Viewed from the medial sagittal section, the locations of Planes 1–7 corresponded to the arched dorsum of the tongue, which ascended gradually in the posterior portion (Fig. 1 P1–P4), peaked in the middle (Fig. 1 P5), and descended towards the anterior tip (Fig. 1 P6–P7). In addition, three anatomical landmarks needed illustration: Plane 1 was the posterior end of the initially emerged PS (Fig. 1 P1); Plane 6 was the transition region, where the tongue began to free from the attachment of the lingual frenulum (Fig. 1 P6); and genioglossus was the fan-shaped extrinsic tongue muscle that forms the majority of the tongue (Fig. 1a, b, empty arrows), whose origin was the mental spine of the mandible and insertions were the hyoid bone and the dorsum of the tongue.^20^ The tongue could be anchored by genioglossal muscles to control mobility.^20^ We further described the morphological interaction between the PS and tongue in the coronal direction at early E12.5 and late E13.5.

### The descending and lateral deviations of the tongue were closely associated with the elevation pattern of the PS along the AP axis

**Fig. 2:**
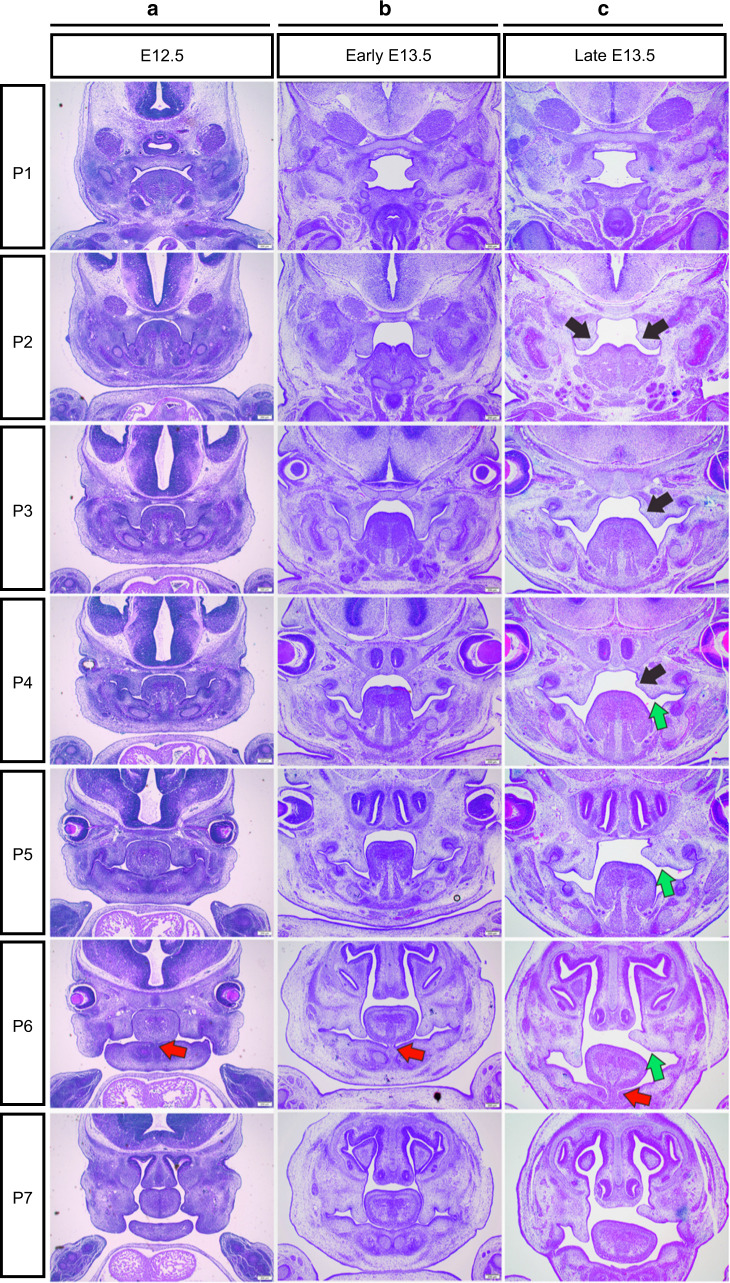
Morphological changes before **a**, **b** and during PS elevation (**c**). The PSs were observed in 7 coronal planes (P1–7) along the AP axis- from E12.5 to late E13.5. P1 was the extremely posterior region where the PSs first emerged and horizontally grew without obstruction from the tongue, implying the inherent spontaneous horizontal growth capacity. P2 and P3 were the posterior, P4 and P5 were the middle, and P6 and P7 were the anterior regions where the PSs were initially vertically constricted between the lateral borders of the mandibular bone and tongue at E12.5 and early E13.5. Black arrows, the new lingual bulging protrusions of PSs; green arrows, the original distal tip of PSs; red arrows, the disappearing frenulum lingualis

It has been reported that defective tongue musculoskeletal attachment correlates with cleft palate, and functional neurotransmission is required for fetal tongue mobility to facilitate palate formation.^13^ In this study, we observed the structural correlation between the palate and tongue before and during elevation. Before elevation (E12.5 and early E13.5), the bilateral PSs grew vertically along the lateral walls of the tongue, and the tongue was erect from the posterior towards the anterior region except for Plane 1, where the palate grew without any interference with the tongue during the whole period (Fig. 2a, b). From the posterior to anterior, the attachment of the genioglossus muscle gradually decreased and eventually disappeared (Fig. 2a, b, P1-P7, the red arrows pointing towards the disappearing frenulum lingualis in Fig. 2 P6). From E12.5 to late E13.5, the descent of the tongue gradually increased throughout the AP axis, and the PS at late E13.5 began to elevate (the black arrows pointing protrusions in Fig. 2c). During elevation, the new protrusion on the lingual wall of the PS reflected the remodeling process in the posterior and middle regions (Fig. 2 P2, P4, black arrows), while there was no such protrusion in the anterior regions (Fig. 2c, P6, P7). Interestingly, the right PS in the anterior region had already completed elevation, while the posterior and middle regions were still undergoing remodeling; this obvious discrepancy was accompanied by the emergence of the lateral deviation of the tongue (Fig. 2c, P6–P7, green arrows). Plane 5 was the transition region where the deviation of the tongue emerged, and the elevation pattern of the PS transformed from slow remodeling to rapid rotation. It was more remarkable that the PS elevated without bulging a new protrusion on the lingual wall in Plane 1, where the tongue was almost invisible (Fig. 2c, P1). The elevation pattern in Plane 1 was that of rapid rotation as the anterior palatal shelves. All together, the above results indicated that the tongue was closely associated with the distinct elevation pattern of the PS along the AP axis.

### Computer-aided 3D reconstruction provided a spatial structural relationship of the palatal shelves and tongue

**Fig. 3:**
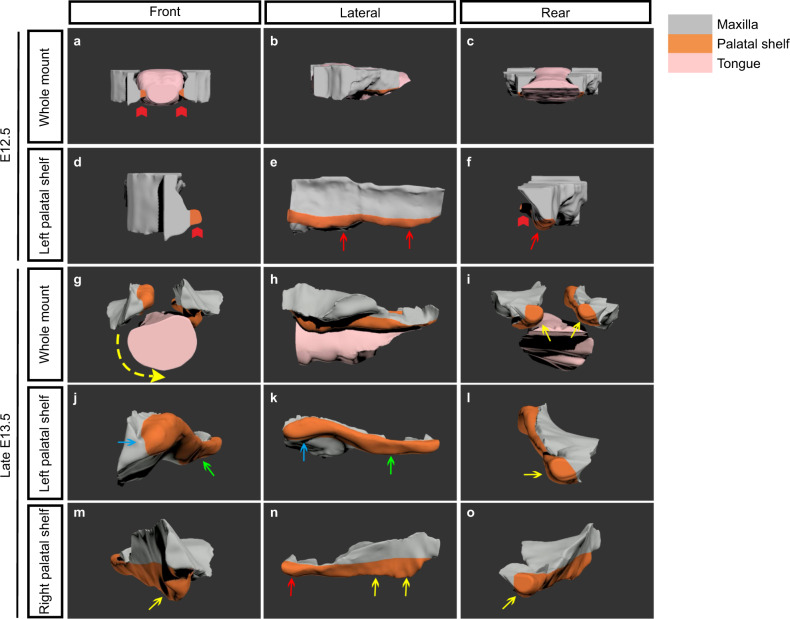
Digital 3D reconstruction of the PSs and tongue at E12.5 and late E13.5. **a**–**c** The whole 3D model of the tongue and PSs, indicating the spatial location relationship at E12.5. **d**–**f** The enlarged single view of the PS at E12.5. **g**–**i** The whole 3D model of the tongue and PSs, showing the spatial location change at late E13.5. **j**–**l** The enlarged single view of the left PS during elevation at late E13.5. **m**–**o** The enlarged single view of the right PS during elevation at late E13.5. red arrowheads in **a**, **d**, and **f**, the anterior PSs growing in a horizontal pattern; red arrows in **e** and **f**, the vertical PS; yellow arrows in **i**, **l**, and **o**, the horizontal growth PSs in the extreme posterior region of PSs; blue arrows in **j** and **k**, the elevated PS in the anterior region of the left PS; green arrows in **j** and **k**, the elevated PS in the middle-posterior region of the left PS; yellow arrows in **m** and **n**, the vertical state of the right PS in the anterior and middle regions; red arrow in **n**, the elevated state of the right PS in the posterior region

To carefully observe the spatial interaction between the PS and tongue during elevation, we developed a digital three-dimensional model of E12.5 and late E13.5 embryos, which was, to our knowledge, innovative in the studies of palatal development (Fig. 3, Supplementary animations 1–5). At E12.5, in the front view of the whole mount and separately enlarged PS models, the anterior part of the PS grew horizontally (Fig. 3a, d, f, red arrows), while the middle and posterior parts of the PSs were absolutely stuck and stretched along the lateral borders in the lateral and rear views by the tongue (Fig. 3e, f, red arrows). During the late E13.5, the anterior part of the left PS completed elevation with an ipsilateral deviation of the tongue, while the right PS was obstructed by the tongue in the vertical direction (Fig. 3g–o). In the lateral view, the anterior region of the left PS had flipped up (Fig. 3j, k, blue arrows), while the middle and posterior regions were still undergoing reorientation (Fig. 3k, green arrows). The right PS initially started elevation by remodeling in the posterior region (Fig. 3n, red arrow), and the middle and anterior regions were still vertical (Fig. 3n, yellow arrows). Thus, different schedules of both sides of PSs again implied that the elevation proceeded from posterior towards anterior, but it was completed first in the anterior region. In the rear view, both sides were growing in a horizontal pattern to avoid tongue obstruction, which suggested the inherent horizontal growth properties of the PSs (Fig. 3i, l, o, yellow arrows).

## Discussion

### Multi-plane observation and digital 3D reconstruction proposed a new perspective on murine PS elevation

Little progress has been made in the past decades regarding the mechanism of the PS elevation because of the narrow time window.^1,4,8^ In this study, we established 7 coronal planes along the AP axis to observe specific morphological features in different palatal regions in detail. The pattern of palatal elevation was proved to be heterogeneous in different regions, as reported by a previous study.^8,21^ An early study suggested that palatal elevation was achieved rostrally by the “flip-up” process from a ventromedial to a horizontal position, while caudally, the outgrowths from the medial surfaces contributed to the process in rats.^22^ A latter study in mice further postulated that posteriorly, the medial border of PSs bulged out whereas their original ventral tip retracted, and the matrix remodeling process via hyaluronate-mediated extracellular matrix swelling played a major role in reorientation of the secondary palatal shelves.^23^ Although the above studies reached a consensus about the regional heterogeneity in PS elevation, there are disputes regarding where the elevation process starts and how it proceeds along the AP axis.^24^ Early researchers showed that the elevation proceeded from the anterior region towards the posterior region in rats,^22^ while a study in mice proved that the posterior region was the starting point.^24^ In the present study, we used more intuitive methods and observed that except for the extreme posterior region of the palate, reorientation proceeded from the posterior region toward the anterior region. Nevertheless, the anterior PS first completed the elevation quickly by the flip-up process while the posterior part still underwent slow remodeling. The regional distinct elevation patterns prompted us to explore decisive factors that contributed to reorientation and rotation of the PS.

### Reorientation in posterior and rotation in anterior were the phenotypes by which the PS proactively overcame the obstruction caused by the tongue occupying the limited oral cavity

During the elevation period (mice embryo, late E13.5), the tongue and palate underwent a vital position interaction.^20,25,26^ Genioglossus is a fan-shaped extrinsic tongue muscle that forms the majority of the tongue. The origin point of genioglossus is the mental spine on the mandible and the end points are the hyoid bone and dorsum of the tongue.^20^ Thereby, with an increase in the length of craniofacial, the tongue could be dropped in a downward direction. A previous study has shown that the downward movement of the tongue played an important role in releasing space during PS elevation.^26–28^ The Pierre Robin Sequence is a congenital condition of facial abnormalities in humans, in which the mandibular bone appears as micrognathia restricting and forcing the tongue to develop upward because of the narrow oral cavity. Consequently, the displaced tongue produced physical obstruction to the PS elevation and resulted in cleft palate.^29^ In addition, the Snail1^−/−^; Snail2^−/−^ mutant mice embryo also developed a phenotype similar to the Pierre Robin Sequence in humans, and the failure of Meckel’s cartilage to extend the mandible led to a small oral cavity preventing proper movements of the tongue and elevation of PSs.^30^ The Bmp7-null embryos generated an abnormal deflection of the genioglossus fibers from the anterior–posterior toward the medial-lateral axis, which altered the correct descent of the tongue and impeded normal palatal elevation.^26^ Thus, it seemed that the tongue was absolutely an obstruction to the normal palatogenesis. For this viewpoint, there were two evidences: a recent study found that the Smoc/c; Hand2-Cre mutant mice exhibited hypoplasia of the tongue, while palatogenesis was completely normal.^12^ Similarly, we also observed spontaneous horizonal growth of the extremely posterior part at E13.5 and anterior part at E12.5 of PS in wide-type mice, where the tongue was relatively small and without interference from the local PS. In relation to the deviation direction, among embryos we have sectioned (data not shown), we collected 15 embryos which showed a lateral deviation. 7 embryos showed left shelf in the vertical direction, right shelf in the horizontal direction, 8 embryos showed a right vertical oriented shelf and a left horizontal oriented shelf. Namely, the percentage of embryos in which there is left deviation is 46.7%, while the percentage of embryos in which there is right deviation is 53.3%, which is not statistically significant. Thus, the tongue could play as an important role in the formation of a distinct elevation pattern along the anterior–posterior axis, and this viewpoint might contribute to investigating the mechanisms regulating PS elevation from a new perspective.

### The advantages and limitations of multi-plane observation and digital 3D reconstruction

With respect to cleft palate, which is one of the most common birth defects, the following two major factors create an obstacle in gaining insights into the molecular mechanisms: rapidly occurring process and palate hidden inside the oral cavity. Currently, the understanding of the mechanism of shelf elevation is mainly based on histomorphological studies from 2D sections and various in vitro studies.^6,7,9,24,31–33^ However, conventional 2D histology provides only planar information of the PS and surrounding tissues. Furthermore, there were no definite sites or anatomical landmarks of the anterior, middle (the presumptive hard palate), and posterior (the presumptive soft palate) regions of the palate, which were inconsistent in various articles and might give rise to misinterpretations and the loss of crucial shelf morphological change during palatal elevation along the AP axis.^24,34^ Dynamic palate culture with the absence of both the tongue and mandible to study the PS elevation ignored the interaction of the PS and tongue, while the examination of the PS elevation with the presence of the tongue and mandible in vitro was not convincing because it could not entirely simulate the in vivo conditions of palate development. To gain comprehensive knowledge about the sequence of PS morphological changes that take place during the elevation process, the 3D histology reconstruction of the entire mouse PS from serial sections is proposed here. Here the new method allowed us the new opportunity to gain insights into the palatal development and the spatial and temporal dynamics adjacent to the mandible and tongue. Normalization of the biological mechanisms regulating palatogenesis in susceptible fetuses is expected to contribute to cleft prevention, pre-natal diagnosis, and possibly in vivo fetal treatment. It may lay the foundation for developing new and better treatment or prevention strategies for cleft palate birth defects. However, there are also some deficiencies of this method. A shortcoming of this method is that it is time-consuming and requires complex pre-processing steps. Before the images were used to create 3D models, hundreds of sections should be stained with HE, and the images have to be captured in sequence. And it is a technically challenging process to cut serial sections by the microtome. Therefore, compared with large tissue mass, it is more suitable for small tissue blocks to reconstruct, which is a limitation for this approach. With our ongoing refinement of image processing and reconstruction technology, high-automation, high-accuracy 3D histology reconstructions will aid in further investigation of the mechanisms regulating palatal shelf elevation.

## Materials and methods

### Animals and desired mice embryo operations

The animal experiments were carried out in strict accordance with the guidelines for the care and use of Laboratory Animal Center of Sichuan University and were approved by the Committee on the Ethics of Animal Experiments of West China Hospital of Stomatology (WCHSIRB-D-2018–118). This study conformed with the guidelines for the use of preclinical animal studies in research.

Eight-week-old male and female C57BL/6J wide-type mice (Laboratory Animal Center of Sichuan University, China) were housed and bred in a specific pathogen-free (SPF) laboratory. They were maintained at 22 °C with a 12 h light/dark cycle, and given a commercial pelleted diet and purified water ad libitum. After mating at 8:00 pm, embryos were noted as 0.5 days post coitum (dpc) according to the day of plug observation. The two developing palatal shelves grow vertically along the lateral sides of the tongue on E12.5 and early E13.5. At late E13.5, the vertical shelves begin to undergo an elevation process, in which the palate shelves become horizontally oriented and move from lateral of the tongue to above the dorsum of the tongue in the oral cavity.

**Fig. 4:**
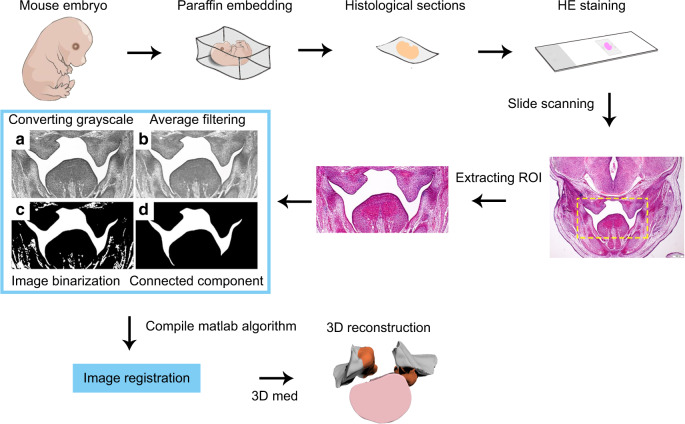
Overall workflow of the digital 3D reconstruction of the palatal shelves and tongue

So we choose accordingly E12.5 and E13.5 as the desired stage to analyze the spatial and temporal changes in general overview of palatal elevation. Three pregnant mice were killed at the each desired stage (E12.5, early E13.5, and late E13.5, during stages E12.5 and E13.5 at 8:00 am to obtain the vertical PS, and during stage E13.5 at 8:00 pm to obtain the elevating PS). For histology, heads were washed in ice-cold PBS (150 mmol·L^−1^ NaCl, 20 mmol·L^−1^ Na-phosphate, pH 7.4) and fixed in 4% paraformaldehyde overnight, dehydrated through a graded series of ethanol, and embedded in paraffin, as described.^24^ Serial histological 7 μm sections were obtained along the anterior–posterior axis (AP axis, sagittal direction), and then stained with hematoxylin and eosin (HE) (Fig. 4). Animal experiments were approved by the ethics committee of West China Hospital of Stomatology, Sichuan University.

### Digital 3D reconstruction of PSs and tongue

The embryos before and under elevation (E12.5 and late E13.5) were serially sectioned into 152 and 250 slices in the coronal direction, respectively, stained with HE, and captured under bright field optics with 4x objectives on an Olympus BX-51 microscope (Aperio Technologies, Vista, CA, USA). These images were saved in TIFF format with 4 080 × 3 072 pixels and grouped as a set of images. All images were saved in the same order as the slice order. In this study, the region of interest was the cavity in the head of the mouse. Since it was impossible to photograph the same position in each section, the region of interest (ROI), whose size is 2 000 × 1 000 pixels, was extracted from each image and aligned (Fig. 4).

During the image acquisition process, noise might be introduced. It interfered with critical image information extraction. To reduce noises in the histology images, the images were firstly converted into greyscale images using the green channel of the RGB color image. Average filtering was then used to filter the greyscale images (Average filtering means for each central pixel; a surrounding 15 × 15 square region was used to calculate its new value. The average gray value of 225 pixels in this region was taken as the filtered gray value. The purpose of this method is to reduce the noise). After filtering, the target is to retrieve the cavity. We chose a threshold of 0.5 to convert the grayscale image into a binary image, divided the image with the value of 1 into many connected components using an 8-neighbor operator, and treated the connected component with the largest oral cavity area (“Connected component” is the operation of extracting the target area after threshold processing). Then we also performed rigid registration of the image so that the target area’s anatomical structure was aligned (Fig. 4). Compile Matlab algorithm” is a simple process. When the Matlab script was written correctly, it can be executed automatically. We are more than happy to share our Matlab code on the Matlab File exchange site, and further details about algorithms and specific approaches are available upon request.

The processed images (Fig. 4), including the palatal shelves and tongue, were imported into 3D Med (3D Medical Image Processing and Analyzing System, Medical Image Processing Laboratory, Institute of Automation, Chinese Academy of Sciences). Using the option of the “Threshold Segmentation and Volume Measurement”, we obtained the initial 3D surface model of a cavity in the mouth of the mouse embryo. The surfaces of the slices were automatically connected. The 3D models were exported with STL format for further design by Geomagic Studio (Geomagic company, Geomagic Studio 2015). Using the “Delete spikes” and “Quick automatic smooth” options, a smoother model was obtained. To facilitate the visualization of the different proportions, the palatal shelves and tongue were marked in orange and pink, respectively, and other parts were marked in gray (Fig. 4). The function “Automatic keyframe animation” was used to create a 3D animation to virtually display the whole model. Finally, 3D animations displayed the PSs and tongue models by 3D Studio Max (Fig. 3). (Autodesk, Max 2015).

## Supplementary information

Polish Language Certificate

Supplementary Animations 1 E12.5

Supplementary Animations 2 E12.5

Supplementary Animations 3 Late E13.5

Supplementary Animations 4 Late E13.5

Supplementary Animations 5 Late E13.5
