# Supplementary material for: Three-dimensional reconstruction of systematic histological sections: application to observations on palatal shelf elevation
Source: Int J Oral Sci. 2021 May 26;13:17. doi: 10.1038/s41368-021-00122-8 (PMC8154959; doi:10.1038/s41368-021-00122-8)
Supplement: Supplementary file 1 — Polish Language Certificate [file 41368_2021_122_MOESM1_ESM.pdf]

Certificate of English Language Editing

Manuscript Title:

3D reconstruction of systematic histological sections: Application to observations on palatal shelf elevation

Date of Revision:

December 24, 2020

Abstract:

Normal mammalian secondary palate development undergoes a series of processes, including palatal shelf (PS) growth, elevation, adhesion and fusion, and palatal bone formation. It has been estimated that more than 90% of isolated cleft palate is caused by defects associated with the elevation process. However, because of the rapidly completed elevation process, the entire process of elevation will never be easy to clarify. In this article, we present a novel method for three-dimensional (3D) reconstruction of thick tissue blocks from two-dimensional (2D) histological sections. We established multiplanar sections…

This document certifies that the manuscript listed above was copy edited for proper English language at LetPub. All of our language editors are native English speakers with long-term experience in editing scientific and technical manuscripts. We are committed to leveling the playing field for researchers whose native language is not English.

- Neither the research content nor the authors' intended meaning were altered in any way during the editing process.
- Documents receiving this certification should be considered ready for publication where language issues are concerned. *However, the authors may accept or reject LetPub's suggestions and changes at their own discretion.*
- If you have any questions or concerns about this edited document, please contact us at support@letpub.com

LetPub Your Manuscript Editor

LetPub is an author service brand owned and operated by Accdon LLC. Headquartered in the Boston area, we are a full-spectrum author services company with a large team of US-based certified language and scientific editors, ISO 17001 accredited translators, and professional scientific illustrators and animators. We advocate ethical publication practices and are an official member of the Committee on Publication Ethics (COPE).

For more information about our company, services, and partnership programs, please visit www.letpub.com.

© 2020 Accdon, LLC. All Rights Reserved. Tel: 1-781-202-9968 Email: info@accdon.com Address: 400 Fifth Ave, Suite 530, Waltham, MA 02451, United States
